# Facial transplantation in emerging adulthood: characterizing challenges and opportunities to support optimal outcomes

**DOI:** 10.3389/frtra.2026.1880820

**Published:** 2026-07-09

**Authors:** Michael E. McKenna, Hailey P. Wyatt, Alexis K. Gursky, Eduardo D. Rodriguez, Laura L. Kimberly

**Affiliations:** 1 Hansjörg Wyss Department of Plastic Surgery, NYU Grossman School of Medicine, New York, NY, United States; 2 Department of Population Health, NYU Grossman School of Medicine, New York, NY, United States

**Keywords:** adherence, cognitive development, emerging adult, face transplant, informed consent, psychosocial support, social integration, treatment decision—making

## Abstract

Ethical considerations in facial transplantation have been widely discussed for more than twenty years, preceding the technical realization of the procedure in 2005. With more than 50 face transplants performed to date in adults worldwide, considerations are evolving beyond risks and benefits toward questions of equity and sustainability. Yet, issues of informed consent, the risk of lifelong immunosuppression, graft lifespan, and metrics for success continue to prompt dialogue, especially for certain patient populations. In this Perspective, we combine our clinical experience with a synthesis of the existing evidence and propose that emerging adults, characterized as individuals aged 18–25, be given particular consideration. Emerging adults occupy a middle ground between adolescence and established adulthood. This formative life stage has a profound impact on an individual's sense of identity and social integration. Emerging adults stand to benefit significantly from facial transplantation precisely because of the key developmental nature of this stage, yet ongoing cognitive development presents challenges for judgement and decision-making that can complicate care for this population and impact outcomes. Given the complexity of facial transplantation and lifelong commitment to follow up required for face transplant recipients, it is critical to understand potential challenges for decision-making and obstacles to adherence that individuals may face in this developmental life stage. Recognizing emerging adults as a patient population with specific support needs will enable face transplant programs to offer tailored supports to improve outcomes for this transformative procedure in emerging adulthood.

## 1 Background

Vascularized Composite Allotransplantation (VCA) is the transfer of a functional unit containing multiple tissue types such as skin, muscle, bone, nerves, and blood vessels (1). While hand transplant is the most common form of VCA, face, uterine, penile, abdominal wall, and larynx transplant have also been performed (2). As a type of VCA, facial transplantation (FTx) is significant for its ability to restore recipients' functional capacity and sense of aesthetic normalcy. The procedure's novelty and profound ethical ramifications have led to extensive media attention since its initiation (3–6). The first successful FTx was performed in 2005 by a team in Amiens, France who replaced the central and lower face of a 38 year old-woman with a facial graft from a brain-dead donor (7). As of 2026, more than 50 face transplants have been performed worldwide (8, 9). The indications for FTx are broad. Severe burns, ballistic injury, motor vehicle trauma, animal attacks, and congenital disfigurements may all lead to profound functional and aesthetic deficits which are not amenable to traditional reconstructive techniques and could be ameliorated by FTx (10, 11). Published reports suggest outcomes of the procedure have been largely positive. For the first 50 FTx recipients, 5 and 10-year survival rates were 85 percent and 74 percent, respectively (8), with many recipients of FTx reporting marked improvements in pain, function, mental wellbeing, and overall quality of life (12–14).

The risks and benefits of FTx for the adult population have been widely discussed, emphasizing the technical aspects of the procedure as well as ethical and psychosocial implications (2). Technical research has focused primarily on the consequences of lifelong immunosuppressive medication, the avoidance of rejection events, and emerging surgical technologies (15, 16). Ethical and psychosocial aspects of FTx include weighing risks and benefits and ensuring robust informed consent, addressing equity in access to care among diverse patient populations, respecting patient privacy, and investigations into geographic and financial barriers to care (2). Ethical concerns in pediatric facial transplantation are the subject of ongoing discussion in the field, with a clearly recognized and distinct set of considerations. Technical questions regarding the feasibility of the procedure remain unanswered, and the ethical implications of FTx in this population, such as capacity to provide informed assent and the risks of long-term immunosuppression and graft loss, are particularly pronounced. To date, FTx has not been performed in children, and the advisability of undertaking pediatric FTx is contested (17, 18). While FTx in adults is less controversial than in pediatric populations, the adult population itself is composed of subgroups with distinct developmental and psychosocial profiles. In this Perspective, we seek to characterize implications of FTx for emerging adults, defined as adults ages 18–25 years old, to inform decision-support needs and strategies to promote optimal outcomes for this distinct population. Drawing on our clinical experience and the literature at the intersection of transplant medicine, developmental psychology, and ethics, we synthesize and apply the existing evidence and suggest preliminary strategies to better support this heretofore understudied group.

## 2 Purpose of FTx: improving quality of life and social integration

FTx is not considered to be a lifesaving surgery in the way that solid organ transplantation (SOT) is generally classified. Patients in need of FTx are not at risk of imminent death, as may be the case for someone in need of a heart or liver transplant. That said, critics have argued that the procedure may still be considered crucial for a patient's quality of life, social integration and wellbeing. FTx has the potential to prevent, or even reverse, the loss of social connection that often accompanies profound facial disfigurement, termed “social death” by scholars in this space (19). Social death encapsulates the sense of isolation, ostracism, and loss of identity felt by many people living with severe facial disfigurement.

While FTx offers significant potential psychosocial benefits related to reduction in social stigma, it is also important to consider the procedure's functional benefits. FTx candidates often present with deficits in their ability to smell, breathe, speak, and experience facial sensation (20). Following FTx, patients report marked improvements in these areas of function. Furthermore, many FTx recipients may achieve independence from life-supporting measures such as tracheostomy and gastrostomy tubes. FTx is also remarkable in its ability to improve speech quality. Many patients with speech deficits are able to regain intelligible speech within a year of transplantation, a key component of social integration (20). The potential of FTx to facilitate social reintegration and ameliorate other significant functional challenges is particularly meaningful when considering FTx in emerging adulthood. Indeed, emerging adulthood's outsized significance in shaping life trajectory may make FTx particularly beneficial for this age group.

## 3 Emerging adults as a distinct population in FTx

### 3.1 Emerging identity and independence

Why consider defining emerging adults as a separate population in the context of FTx? Emerging adulthood encompasses individuals in the range of 18–25 years old. Although emerging adults share traits with adolescents and adults, developmental psychology has come to view this population as a distinct group based on the work of Jeffrey Arnett, who is credited with developing the term in 2000 (21). The basis for this distinction is attributed to the fact that emerging adults have not yet assumed many of the responsibilities of typical adulthood but have largely moved beyond the restrictive social structures and oversight of adolescence. As a result, emerging adults are capable of exploring their identities with a freedom and independence that is generally not possible at other stages of life (21). In previous decades, the distinction between emerging adults and young adults may have seemed trivial. However, the psychosocial factors precipitating the emergence of this new group—particularly in industrialized and technology centered societies—have become more pronounced in recent years.

Marriage, family life, and career building are traditionally thought to be the defining steps in the transition from adolescence to adulthood (22). Yet young people in the United States are marrying at later ages (23). Furthermore, from 1970 to 2014, the average maternal age at first childbirth rose from 21.4 years to 26.3 years and the average paternal age at first childbirth rose from 27.4 years to 30.9 years (24). As 18–25-year-olds are settling into the typical roles of adulthood later in life, this shift in responsibility and attitude has opened new opportunities for this age group. Having left the dependency of childhood and adolescence, emerging adults find themselves at a position in life with an extraordinarily broad scope of possibilities—most notably in the domains of romantic attachments, establishment of livelihood, and the formation of their worldviews (21). This window of opportunity becomes especially relevant when treating emerging adults in a medical setting. A condition such as sudden, profound facial disfigurement, may severely limit these areas of opportunity during a critical formative window. Consideration of the psychosocial makeup of this group is paramount to ensuring the support emerging adults may need when receiving medical care.

### 3.2 Developing cognition

Arnett's conceptual designation of emerging adulthood as a specific developmental phase has gained widespread traction in developmental psychology and is further supported by advances in cognitive neuroscience (25–27). For example, risk taking behavior in emerging adults results in a markedly higher mortality risk when compared to other populations. This likely reflects a combination of emerging adults' freedom and their incomplete brain maturity (28). It is well known that the prefrontal cortex serves as the nidus for executive function, allowing for abilities such as planning, abstract thinking, emotional control, and coping with stress (29). However, the growth of brain structures does not occur simultaneously. While the prefrontal cortex generally matures into the mid- to late 20s, growth of the reward-driven striatum, medial, and orbital prefrontal cortices occurs earlier than the regions responsible for cognitive control. The resultant discrepancy as explained by the, “dual-system model,” is often cited as a source of the higher rates of impulsivity, sensation seeking, and risky decision-making that characterizes young and emerging adulthood (30). While emerging adults are by no means incapable of making difficult and nuanced decisions, considering their developmental stage is important when evaluating the ethical ramifications of a life-altering procedure such as FTx, since the procedure carries long-term risks which may be harder for an emerging adult who has not reached full cognitive maturity to understand, appreciate, and integrate into their decision-making process.

### 3.3 Insights from SOT

Surgical areas such as SOT can provide valuable insights into the particular support needs this patient population may experience. As in FTx, the success of SOT relies on long-term adherence to immunosuppressive medication. For kidney transplants, older adolescents and young adults (a cohort defined as 17–24 year olds), irrespective of the age at transplant, were found to have the highest risk of graft failure (28). The consequences of nonadherence and its associated risk of graft failure have the potential to reduce life expectancy, opportunities for repeat transplantation, and success in work and school (31). Factors influencing nonadherence in SOT are broad, but most are rooted in the fact that regions of the brain mature on differing timelines (32–34).

Medication adherence is an important issue in FTx and research from SOT is valuable for understanding this dimension of risk in FTx. However, the indications and outcomes for FTx differ substantially from SOT. As an externally visible procedure with significant aesthetic as well as functional indications, FTx involves ethical and psychosocial issues beyond the scope of SOT. In this sense, FTx closely aligns with reconstructive plastic surgery procedures treating other forms of facial disfigurement. In general, congenital disfigurements are more likely to have improved outcomes when corrected during childhood and adolescence (35–37). Although not typically performed in emerging adults, the early age at which these reconstructive surgeries typically take place reflects a desire not only to restore function but also to address and hopefully mitigate psychosocial disadvantages and biases that individuals with significant visible facial differences may experience across the life course.

### 3.4 Implications for informed consent

Fully informed consent reflects an ongoing process of shared decision making between the surgical team and the patient (38, 39). Emerging adults are legally considered adults capable of providing informed consent, which differentiates them from adolescent and younger pediatric populations. Advances in neuroscience suggest that cognitive development continues through the mid-twenties, and the rate at which emerging adults mature cognitively during this time period varies between individuals (40, 41). Care should be taken to ensure that emerging adult FTx candidates are fully informed and demonstrate the judgement needed to make reasonable decisions about short and long-term risks and requirements of the procedure, particularly given the lifelong nature of follow up needed to maintain a functioning graft. Recognizing that emerging adulthood is still a time of flux for the maturing brain provides an opportunity for care teams to tailor the consent process to better promote optimal outcomes within this population.

### 3.5 Fully understanding risks of morbidity and mortality in FTx

Following FTx, immunosuppressive medication is essential to the maintenance of the graft. Emerging adults are at a heightened risk of struggling to adhere to medication regimens. For the first time in their lives, many emerging adults are living outside of the scrutiny of parental oversight and social structures such as school. Unlike in SOT, where retransplantation is not uncommon, retransplantation in FTx is rare. To date, only two facial retransplantations have been performed, both of which were in individuals past their 40s (42, 43). Such data further underscores the importance of medication adherence. The threat of irreplaceable graft failure is a serious consideration in FTx.

Immunosuppressive medications needed to maintain graft function carry their own serious risk profile, and FTx teams must strike a fine balance between under and over-immunosuppression. With too little immunosuppression, patients are at risk of losing the graft. With too much immunosuppression, the probability of opportunistic bacterial, fungal, and protozoal infection all increase (44–46). Furthermore, posttransplant malignancies are well known potential consequences of lifelong immunosuppression. While strategies have been developed to reduce the nonimmunologic risks associated with immunosuppressive medications, immunosuppressive medications are accompanied by a host of potential deleterious effects on cardiovascular, renal, endocrine, gastrointestinal, and musculoskeletal health, including organ toxicity (47–52). While established adults must also accept the risks associated with immunosuppressive medication, the earlier age at which EAs receive their graft means greater lifetime exposure to the harmful effects of the medications.

Ultimately, the toxicity of long-term immunosuppressive medication may lead to irreversible damage resulting in death; the real possibility of a shortened lifespan looms, particularly for emerging adults who are otherwise in good health and would have a typical lifespan ahead of them. Furthermore, emerging adults usually have little if any experience making a decision to pursue a life-altering procedure with possible risk of mortality. They are at a life stage where they are likely still developing their thoughts on death and dying (53). Thus, they may be undertaking a procedure where a full understanding of the existential consequences may only be apparent with hindsight. The chance of post-operative regret is possible, and special care must be taken in this population to ensure that they have a thorough understanding of the long-term complications of FTx. Indeed, the capacity to fully understand the long term consequences of one's actions is crucial to the consenting process when undertaking major medical procedures, especially for those, such as FTx, that are considered elective (54). This is a major consideration when taking into account the greater likelihood of a shortened lifespan with FTx (2).

### 3.6 Beyond functional benefits: milestones and identity formation

FTx recipients report marked improvements in function. The functional gains of the procedure work synergistically with the aesthetic benefits to enhance FTx recipients' social integration in a number of domains. Focusing on the framework of emerging adulthood, particularly the significance of this stage in reaching milestones in romantic attachment, livelihood, and worldviews, it is not surprising that FTx offers benefits in social integration and personal development. While FTx can be a life-altering event for established adults, their progression through social milestones places them in a position of relative stability compared to a younger person, for whom choices about livelihood or romantic partnering remain more open and subject to change.

Identity exploration is one of, if not the most, important drivers for the consideration of FTx in emerging adults. In largely individualistic, Western contexts, the beginning of the search for a sense of self results from a combination of (a) increased mental capacities to consider multiple possibilities and (b) a social context where multiple alternatives are available and from which one or more may be selected (55). The active consideration of identity alternatives is assumed to subside once one has enacted firm adult commitments (however “adult commitments” are defined in the society where one lives) and has entered into adult roles such as permanent partnership, employment, and parenthood (56).

Leaving adolescence, emerging adults find themselves in a social context where multiple opportunities exist for the direction of their lives. Crucially, they now possess the mental capacity to rationally consider such opportunities (57). Identity formation is a lifelong process, but the latitude of freedom enjoyed by emerging adults affords them the chance to make choices with uniquely profound consequences on their inner and outer lives. A robust social context and support system is essential during this window of opportunity (58). The possibility of social reintegration is perhaps the greatest benefit FTx can offer emerging adults during a key phase. In facilitating a greater sense of social integration, FTx affords emerging adults the opportunity to experiment with and solidify their identities in areas such as sexuality and physical intimacy, religion, life philosophy, and career choice (59).

## 4 Tailoring approaches to meet support needs

Engaging emerging adults in a robust shared decision-making process is essential, with particular attention to understanding short- and long-term risks and benefits, including challenges with adherence and the limits of current knowledge. Peer-recipient counseling and comprehension techniques such as the teach-back method have already been demonstrated to be effective in other areas surgery and transplantation (60, 61). Applying such techniques in tandem with adherence support planning and long-term mental health, employment, and social reintegration follow up has the potential to promote wellbeing and improve outcomes in emerging adult FTx recipients (13, 62–64).

## 5 Conclusion

FTx is a highly innovative procedure requiring rigorous rehabilitation and a lifelong commitment to follow up care for recipients. Occupying space at the forefront of innovative medicine, significant resources are dedicated to each FTx and special care is given to monitoring and supporting the success of FTx recipients. Emerging adults, whose identities are still evolving, may benefit in particular from the social reintegration FTx can facilitate, yet this life phase also introduces challenges for judgement and long-term adherence. Given the complexity of FTx and lifelong commitment to follow up required for FTx recipients, it is critical to understand issues for decision-making and obstacles to adherence that individuals may face in this developmental life stage. Surgical teams should be aware of the psychosocial factors that may influence their patients' motivations and decision-making processes, and attendant ethical implications for pre- and post-transplant evaluation and support. Emerging adulthood is a transformative period, and FTx during these years has the potential to be a profoundly life-altering experience.

## Data Availability

The original contributions presented in the study are included in the article/Supplementary Material, further inquiries can be directed to the corresponding author.
